# Case Report: Preoperative embolization for a large intracranial pheochromocytoma metastasis: a novel approach to minimize intraoperative risk

**DOI:** 10.3389/fonc.2026.1848791

**Published:** 2026-07-14

**Authors:** Lloyd Mulenga Mwibwe, Qing Mao, Tao Chang, Yixuan Zong, Yuxin Quan, Yu Li, Yuan Fang, Liyang Zhang, Qicheng Shu, Siliang Chen, Yanhui Liu, Bai Xue, Fu Min, Yuan Yang

**Affiliations:** 1 Department of Neurosurgery, West China Hospital, Sichuan University, Chengdu, Sichuan, China; 2 Department of Critical Care Medicine, West China Hospital, Sichuan University, Chengdu, Sichuan, China; 3 Department of Anesthesiology, West China Hospital of Sichuan University, Chengdu, Sichuan, China

**Keywords:** alpha-adrenergic blockade, catecholamine-secreting tumor, embolization, metastasis, pheochromocytoma

## Abstract

Intracranial metastasis from pheochromocytoma is exceedingly rare, with fewer than 100 cases reported in the literature. While preoperative embolization is established for extracranial pheochromocytoma metastases, no prior reports describe its use in the intracranial setting. We present a 65-year-old woman with known metastatic pheochromocytoma who developed progressive right hemiparesis and dysarthria from a 7.8 × 6.0 cm hypervascular left parietal mass with multi-compartmental involvement and calvarial erosion, representing one of the largest and most anatomically complex intracranial pheochromocytoma metastases reported. Markedly elevated plasma metanephrines confirmed functional disease, and multidisciplinary planning identified dual risks of catastrophic hemorrhage and catecholamine-mediated hemodynamic instability. We therefore performed superselective transarterial embolization prior to resection, the first reported use of this technique for an intracranial pheochromocytoma metastasis, achieving near-complete devascularization. Gross total resection was accomplished without hypertensive crisis, with postoperative normalization of catecholamines and significant neurological improvement. This case demonstrates that preoperative embolization offers a dual benefit, reducing intraoperative hemorrhage and attenuating catecholamine release, and should be considered for all functional intracranial pheochromocytoma metastases regardless of tumor size or degree of vascularity.

## Introduction

Pheochromocytomas are catecholamine-secreting neuroendocrine tumors that arise from chromaffin cells of the adrenal medulla (1–5). Malignant pheochromocytoma is defined by metastatic spread to sites devoid of chromaffin tissue (6–8). However, the presence of these tumors in unusual locations, such as the lung or liver, is not necessarily diagnostic of metastasis, as these may represent primary sites (19). Intracranial metastasis is exceedingly rare, with fewer than 100 cases reported in the literature over the past decades (9).

Surgical resection remains the primary treatment modality for pheochromocytomas, but tumor manipulation can precipitate excessive catecholamine release, causing significant hemodynamic instability (10, 11). In severe cases, this can lead to hypertensive crises and potential complications such as myocardial infarction or stroke (10, 12, 13). Preoperative embolization of arterial feeders has been shown to reduce both intraoperative blood loss and catecholamine-mediated hemodynamic instability during resection of metastatic pheochromocytoma (14–16). However, there are no prior reports of this technique being employed in cranial metastasis.

We present the first documented case of preoperative embolization for an intracranial pheochromocytoma metastasis. At 7.8 × 6.0 cm with multi-compartmental involvement and calvarial erosion, this tumor represents one of the largest and most anatomically complex intracranial pheochromocytoma metastases successfully resected.

## Case description

### Clinical presentation

A 65-year-old woman with a history of pheochromocytoma and metastatic disease to the liver was discovered to have a 7.8×6.0 cm left parietal mass on surveillance 68Ga-DOTATATE PET-CT in October 2025. No additional DOTATATE-avid lesions were identified at that time. At the time of discovery, the patient was asymptomatic. Three weeks later, she developed progressive right-sided weakness and dysarthria. She then presented to our neurosurgery department in November 2025.The patient did not report classical paroxysmal catecholamine-related symptoms such as episodic headache, palpitations, diaphoresis, tremors, or paroxysmal hypertension. On physical examination upon presentation to neurosurgery, the patient was alert and oriented. Neurological examination revealed dysarthria and right-sided hemiparesis (right arm 4/5, right leg 3/5). Her past medical history was also significant for hypertension and diabetes mellitus.

The patient had a history of adrenal pheochromocytoma that was discovered when she presented to an outside hospital and underwent right adrenalectomy in July 2018. In May 2023, she underwent resection of a hepatic metastasis. Hepatic recurrence was confirmed in August 2025. The patient had no family history of multiple endocrine neoplasia (MEN) and Von Hippel–Lindau (VHL) syndrome.

### Imaging studies

Upon admission to our neurosurgical unit, comprehensive neuroimaging was performed (**Figure 1**). Non-contrast head CT demonstrated a large, extra-axial hyperdense left parietal mass with significant mass effect and irregular calvarial erosion. MRI demonstrated an irregular, lobulated extra-axial mass in the left parietal region measuring 7.8 × 6.0 cm in maximal cross-sectional dimension. The lesion appeared isointense on T1-weighted imaging and slightly hyperitense on T2-weighted imaging. Following contrast administration, the mass demonstrated marked, heterogeneous enhancement with numerous dilated, tortuous, and disorganized intratumoral vessels. Significant mass effect was present, with compression of the adjacent brain parenchyma, narrowing of the left lateral ventricle, and surrounding patchy vasogenic edema. Mild rightward midline shift was noted. The lesion exhibited broad dural contact with associated dural thickening and an arcuate dural configuration. Focal osteolytic erosion of the adjacent calvarium was evident, with partial extension of the mass through the cranial defect into the overlying left parietal subgaleal soft tissues.

**Figure 1:**
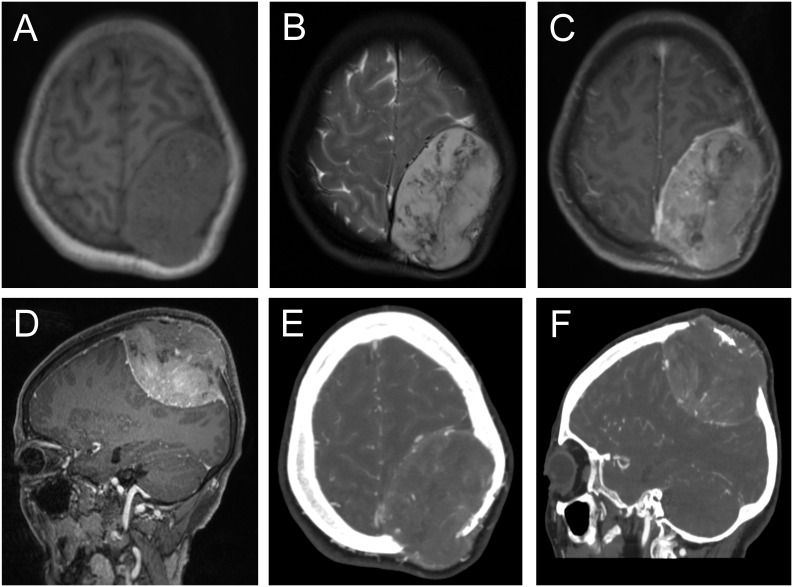
Preoperative images demonstrating a 7.8 × 6.0 cm left parietal lesion on axial T1 MRI **(A)**, axial T2 MRI **(B)**, axial T1 with contrast **(C)**, sagittal T1 with contrast **(D)**. Axial CT **(E)** and sagittal CT **(F)** demonstrating calvarial erosion.

### Biochemical testing and multidisciplinary planning

Biochemical testing demonstrated markedly elevated plasma catecholamine and metanephrine levels: epinephrine 2.86 nmol/L (reference < 0.34 nmol/L), metanephrine 31.63 nmol/L (reference < 0.42 nmol/L), and normetanephrine 17.77 nmol/L (reference < 0.71 nmol/L). Based on the patients past medical history, imaging and biochemical testing results, metastatic pheochromocytoma was suspected. A multidisciplinary team (MDT) involving Neurosurgery, Endocrinology, Urology, Anesthesiology, and Cardiology was convened. Consensus was reached to initiate preoperative alpha-adrenergic blockade with phenoxybenzamine to ensure the patient was hemodynamically stable for surgery. The team also agreed on angiography and subsequent embolization to minimize intraoperative blood loss.

### Angiography and preoperative embolization

Cerebral angiography demonstrated a left parietal tumor supplied predominantly by branches of the left external carotid artery, specifically the left middle meningeal and occipital arteries, with a minor contribution from the right external carotid artery (**Figure 2**).The patient underwent transarterial embolization under local anesthesia using lidocaine without conscious sedation or general anesthesia. Standard peri-procedural monitoring, including continuous blood pressure, heart rate, and oxygen saturation monitoring, was maintained throughout the procedure. Superselective embolization of the left middle meningeal and occipital artery feeders was performed using polyvinyl alcohol (PVA) particles, achieving near-complete angiographic devascularization.

**Figure 2:**
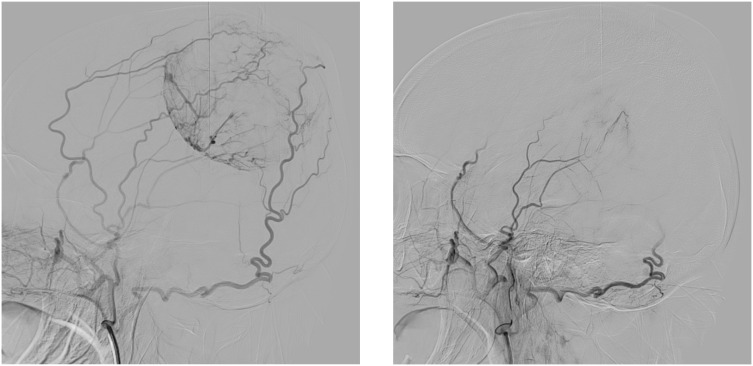
Angiographic images demonstrating tumor blood supply vessels before (left) and after (right) embolization.

### Perioperative and anesthetic management

Perioperative concerns centered on the risk of catecholamine-induced hypertensive crises during tumor manipulation and post-resection hypotension. Preoperative blood pressure optimization was undertaken under multidisciplinary supervision. Alpha-adrenergic blockade with phenoxybenzamine was initiated and titrated prior to surgery. Metoprolol was subsequently introduced after adequate alpha blockade had been established to control reflex tachycardia. Blood pressure and heart rate were monitored three times daily throughout hospitalization. Diabetes mellitus was managed with insulin aspart before meals, insulin glargine at bedtime, and blood glucose monitoring performed four times daily. Given the significant vasogenic edema and mass effect demonstrated on neuroimaging, the patient received dexamethasone for cerebral edema management and mannitol for intracranial pressure control. Seizure prophylaxis was administered with levetiracetam.

To minimize sympathetic stimulation and attenuate the hemodynamic response to direct laryngoscopy and endotracheal intubation, anesthetic induction utilized an opioid-based technique with intermittent nicardipine administration. Invasive arterial and central venous pressure monitoring was established to allow continuous hemodynamic assessment throughout the procedure. A full suite of vasoactive medications, including nicardipine, esmolol, phentolamine, and sodium nitroprusside, was prepared at the bedside to permit rapid treatment of catecholamine-mediated hemodynamic instability during tumor manipulation. Despite the tumor’s size, hypervascularity, and documented secretory activity, no hypertensive crises or significant hemodynamic instability occurred during tumor manipulation.

### Surgical resection

On December 1^st^ 2025, the patient underwent surgery for tumor resection under general anesthesia. The patient was positioned in the lateral decubitus position and a curvilinear incision was made. An abnormal scalp protrusion was observed over the left parietal region. Following elevation of the scalp flap, tumor erosion through the calvarium with extension into the overlying scalp was evident. The mass was tightly attached to and infiltrating the underlying bone. A craniectomy encompassing the involved bone was performed to completely expose the lesion. The main tumor mass was located in the left parietal region, exhibiting a moderately firm, partially solid consistency with rich vascularity. The lesion occupied epidural, dural, and subdural compartments. Critically, although the interface with the adjacent brain parenchyma appeared poorly defined, there was no clear evidence of cortical invasion, and a dissection plane was maintained. The tumor and surrounding involved dura were completely resected, and no remaining mass was seen in the subdural space or invading the brain parenchyma (**Figure 3**). Following resection, the dural defect was repaired with a collagen matrix graft and the cranial defect was reconstructed with a synthetic cranioplasty secured with titanium fixation. Estimated blood loss was 1000 mL. The patient was transfused with 3.5 units of packed red blood cells and 800 mL of fresh frozen plasma. Notably, no hypertensive crises or hemodynamic instability occurred during tumor manipulation.

**Figure 3:**
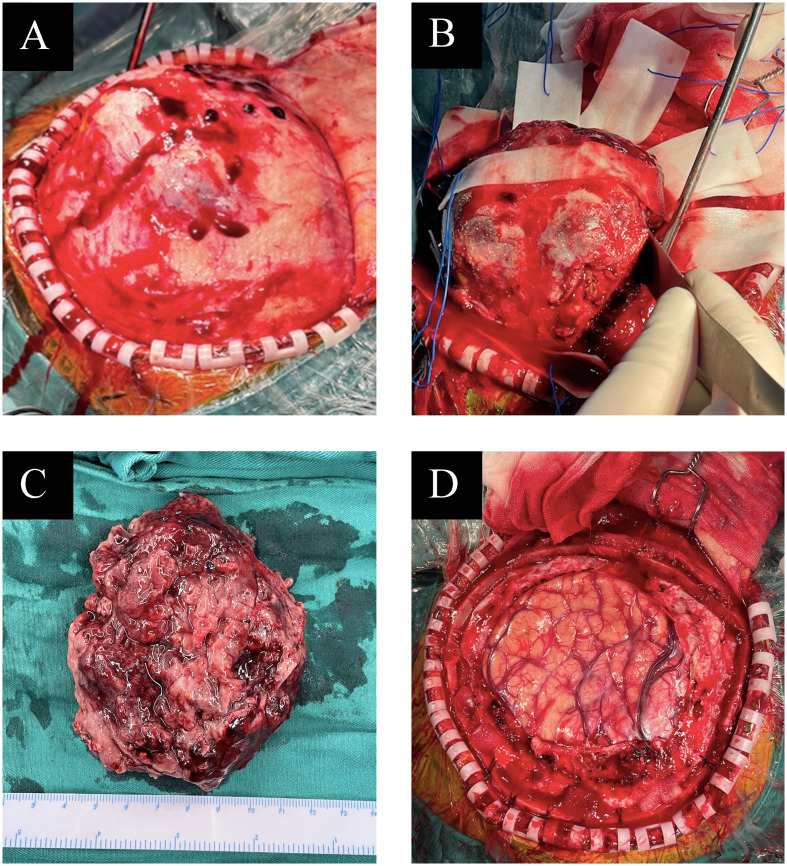
Intraoperative images demonstrating tumor erosion through the calvarium **(A)**. A craniectomy encompassing the involved bone was performed with subsequent careful dissection of the tumor from underlying brain tissue **(B)**. The moderately firm and richly vascular tumor was resected **(C)** no visible invasion of underlying brain parenchyma was observed **(D)**.

### Pathological findings

Histopathological examination demonstrated neoplastic cells arranged in nested and trabecular architectures, encompassed by a complex network of capillaries. The tumor cells exhibited round-to-oval nuclei with distinct nucleoli and granular cytoplasm. Immunohistochemical analysis was positive for chromogranin A, synaptophysin, and succinate dehydrogenase subunit B (SDHB). Staining was negative for cytokeratin (Pan-CK), S100, inhibin, steroidogenic factor-1 (SF-1), and thyroid transcription factor-1 (TTF-1). The Ki-67 (MIB-1) proliferation index was approximately 8%.

### Postoperative course

Immediately following surgery, the patient was transferred to the neurosurgical intensive care unit. By postoperative day 3, her right-sided weakness and dysarthria had begun to improve. On postoperative day 4, plasma epinephrine, metanephrine, and normetanephrine levels had normalized to 0.75 nmol/L, 1.49 nmol/L, and 0.80 nmol/L, respectively. Follow-up MRI confirmed gross total resection with no residual tumor (**Figure 4**). She was discharged on postoperative day 7 in stable condition, with improved neurological function.

**Figure 4:**
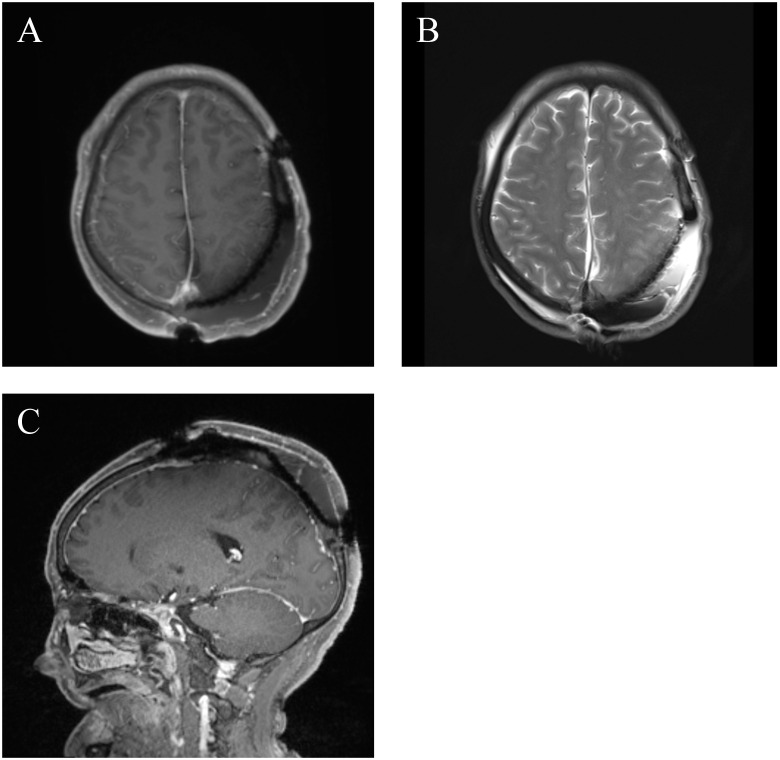
Postoperative images demonstrating gross total resection of the tumor on axial T1 MRI with contrast **(A)**, axial T2 FLAIR MRI **(B)** and sagittal T1 MRI with contrast **(C)**.

### Follow up

At discharge, a structured surveillance strategy was established. The patient was referred to Neurosurgery, Oncology, and Endocrinology outpatient clinics for continued management. Contrast-enhanced brain MRI and plasma catecholamine measurements were scheduled within 1–3 months postoperatively, with subsequent surveillance testing and imaging performed at 3–6 month intervals based on clinical status and radiographic findings. Ongoing blood pressure monitoring was recommended, and Endocrinology advised transitioning from phenoxybenzamine to a calcium channel blocker or angiotensin receptor blocker for long-term antihypertensive therapy. Following multidisciplinary discussion, adjuvant radiotherapy was not pursued because there is currently no established evidence supporting routine postoperative irradiation after complete resection of a solitary intracranial pheochromocytoma metastasis.

## Discussion

This case presented multiple converging risk factors for intraoperative hemodynamic instability. With a 7.8 × 6.0 cm tumor—well above the 4 cm threshold associated with hemodynamic complications—and plasma metanephrine levels exceeding 30 nmol/L, our patient met established criteria for severe intraoperative lability (14, 17). The intracranial location added further complexity: even transient hypertensive surges could precipitate intracranial hemorrhage, while profound post-resection hypotension could compromise cerebral perfusion. Multi-compartmental involvement with calvarial erosion suggested that extensive surgical manipulation would be required for complete resection.

### Biochemical and imaging evaluation

Our patient’s markedly elevated metanephrine (31.63 nmol/L) and normetanephrine (17.77 nmol/L) confirmed functional metastatic pheochromocytoma and guided our aggressive preoperative strategy. However, as Chen et al. demonstrated, normal catecholamine levels do not exclude intracranial disease, with their own patient presenting with normal 24-hour urinary metanephrines despite a large temporal metastasis (18). This suggests that biochemical screening alone cannot exclude intracranial disease in patients with known metastatic pheochromocytoma (18).

Functional imaging plays a critical role in detecting metastatic pheochromocytoma (1). In our case, surveillance ^68^Ga-DOTATATE PET-CT incidentally detected the intracranial lesion before neurological symptoms developed, demonstrating the value of functional imaging for early detection that might be missed on conventional anatomical imaging alone. Comprehensive MRI, which is preferred over CT in metastatic disease for better delineating tumor relationships to adjacent parenchyma (1), revealed a 7.8 × 6.0 cm hypervascular mass with multi-compartmental involvement, calvarial erosion, and marked mass effect. Combined with heterogeneous hyperperfusion on perfusion-weighted imaging, these findings indicated an extremely vascular lesion requiring aggressive devascularization. Digital subtraction angiography confirmed predominantly external carotid artery supply via the middle meningeal and occipital arteries, guiding our targeted embolization strategy.

### Genetic testing

The 2022 WHO classification of endocrine tumors now mandates integration of molecular biomarkers into the diagnostic workup of all PPGLs (19). The molecular classification of pheochromocytomas and paragangliomas (PPGLs) into genomic clusters provides critical prognostic context. Cluster 1 (pseudohypoxic) tumors, driven predominantly by mutations in SDHx genes (SDHA, SDHB, SDHC, SDHD, SDHAF2) or VHL, confer the highest individual metastatic risk and are present in 30–40% of patients with confirmed metastatic PPGL (20, 21). In contrast, Cluster 2 tumors (kinase signaling; RET, NF1, TMEM127, MAX) and the recently described Cluster 3 (WNT-altered) demonstrate lower but non-negligible metastatic potential (20). In this study, Immunohistochemical analysis demonstrated retained (positive) SDHB expression in the tumor cells. This suggests that an SDHx/Cluster 1-driven etiology less is probable, and is more consistent with a non-SDHx molecular background, including Cluster 2 variants (RET, NF1, TMEM127, MAX) or sporadic disease. Mutations in any SDHx gene result in absent or markedly reduced SDHB staining on immunohistochemistry; conversely, retained SDHB expression indicates structural preservation of the complex and argues against a pathogenic SDHx mutation (22, 23). It should be noted however that metastatic pheochromocytoma occurs across all molecular subtypes; retained SDHB expression does not exclude metastatic potential, but rather indicates that the metastatic biology in this case likely arises through mechanisms other than SDH deficiency (20, 22).

### Multidisciplinary coordination: a critical component

The complexity of this case necessitated early and structured multidisciplinary involvement. Our multidisciplinary team comprising neurosurgery, endocrinology, anesthesiology, interventional neuroradiology, and cardiology met formally after imaging and biochemical confirmation. Together, we coordinated the management strategy.

In accordance with Endocrine Society guidelines, we initiated alpha-adrenergic blockade with phenoxybenzamine 10 days preoperatively, titrated to achieve steady blood pressure control, followed by metoprolol after adequate alpha-blockade was established to manage reflex tachycardia (1). Liberal sodium and fluid intake was encouraged to reverse catecholamine-induced blood volume contraction and prevent postoperative hypotension (1). Chen et al. emphasized the critical nature of this preparation, noting that anesthetic agents, surgical manipulation, and even routine medications can trigger massive catecholamine release with resultant blood pressure fluctuations and arrhythmias (18).

Postoperatively an extensive review of adjuvant therapy in intracranial pheochromocytoma metastasis revealed mixed results, informing our decision to withhold postoperative treatment. While Kharlip et al. and Cho et al. demonstrated favorable responses to radiotherapy in their respective cases (24, 25), Miyahara et al. and Kammoun et al. reported poor results with both radiotherapy and stereotactic radiosurgery (26, 27), and no consistent benefit attributable to adjuvant irradiation after complete resection has been established. Regarding systemic chemotherapy, the CVD regiment has been used in malignant pheochromocytoma, but among the 17 reviewed cases only 3 patients received chemotherapy (24, 28, 29), generally limited to unresectable or progressive disease. Given confirmed gross total resection, absence of residual disease on postoperative MRI, and the lack of evidence supporting routine adjuvant treatment in this setting, a surveillance strategy with serial neuroimaging was adopted.

### The decision for preoperative embolization

The tumor posed dual risks: substantial intraoperative hemorrhage from its size and anatomical complexity, and catecholamine-mediated hemodynamic crises from its functional activity (markedly elevated plasma catecholamines). Given these risks, our multidisciplinary team made a critical decision to pursue preoperative embolization despite the absence of prior reports in intracranial pheochromocytoma metastases. This decision was informed by emerging evidence from extracranial sites. Di Daniele et al. demonstrated that embolization of a large pelvic paraganglioma produced a 60% reduction in plasma chromogranin A and 72% reduction in urinary vanillylmandelic acid, suggesting that devascularization directly reduces the tumor’s secretory capacity (30). More recently, Singh et al. reported successful embolization of a cervical spine metastasis using liquid embolic agent (Onyx), achieving complete devascularization without catecholamine release during subsequent resection and only 500 mL blood loss (31). Kaloostian et al. similarly demonstrated that embolization facilitated en bloc spinal resection and metabolic cure (14). Even Boettcher et al., after experiencing 3000 mL blood loss and labile blood pressure during resection of an unembolized intracranial metastasis, explicitly recommended preoperative embolization for these hypervascular tumors (32).

We proceeded with superselective embolization of the middle meningeal and occipital artery feeders using PVA particles, achieving near-complete angiographic devascularization. We selected PVA particles over liquid embolic agents based on our institutional experience and the external carotid artery supply pattern, though future intracranial cases might benefit from the more durable embolization and better visualization offered by liquid agents, as Singh et al. noted in their cervical spine case (31).

### Observations

Despite the high-risk profile, our patient remained hemodynamically stable throughout the entire resection, with no hypertensive crises during tumor manipulation. Estimated blood loss was 1000 mL, substantial but manageable and considerably less than the 3000 mL reported by Boettcher et al. in their unembolized case (32). Notably, their tumor was smaller and purely epidural (32).

This represents the first documented use of preoperative embolization for intracranial pheochromocytoma metastasis. While embolization has been successfully employed for pelvic and spinal pheochromocytoma metastases, no prior reports describe its application in intracranial metastasis (14, 30, 31). Although we cannot definitively isolate the contribution of embolization in a single case, the convergence of our results with the biochemical evidence from Di Daniele et al. and clinical outcomes from Singh et al. and Kaloostian et al. suggests that embolization reduced both the vascular supply and the perfused secretory tissue mass (14, 30, 31). This dual benefit of reduced bleeding and reduced catecholamine surge potential may be particularly valuable in the intracranial setting where the consequences of either complication are more dire.

### Lessons

While the Endocrine Society guidelines recommend alpha-adrenergic blockade for preoperative patient optimization (1), outcomes with pharmacologic preparation alone remain variable. Newell et al. and Boutros et al. demonstrated that adrenergic blockade does not consistently prevent severe intraoperative hypertension, tachycardia, or ventricular arrhythmias (11, 33). In intracranial metastases specifically, Boettcher et al. experienced profound hemodynamic instability despite adequate blockade (32), while Fercho et al. reported stable conditions (9). This variability suggests that factors beyond pharmacologic preparation influence outcomes. Furthermore, preoperative embolization is traditionally indicated for hypervascular intracranial lesions to minimize intraoperative hemorrhage (34). In pheochromocytomas however, embolization may offer additional benefit by reducing not only tumor vascularity but also the perfused secretory tissue mass capable of catecholamine release during surgical manipulation (14, 30, 31). Preoperative embolization should therefore be considered for functional intracranial pheochromocytoma metastases regardless of tumor size or degree of vascularity. The decision framework should account for the tumors biochemical activity and institutional expertise in both embolization and perioperative management of these complex lesions.

Several technical considerations merit discussion. First, the timing of embolization (performed 24 to 48 hours prior to resection) allowed for planning while minimizing the risk of collateral revascularization. Second, although Singh et al. advocated for liquid embolic agents citing more durable embolization and better visualization in their cervical spine case (31), we achieved effective and sustained devascularization with PVA particles in the intracranial setting. Future studies should evaluate the efficacy and safety of PVA particles versus liquid embolic agents for intracranial pheochromocytoma embolization. Third, distal catheter positioning within feeding vessels proved critical to safe embolization. We advanced catheters as far distally as possible within middle meningeal and occipital branches to ensure direct tumor penetration and avoid reflux into collateral pathways. Singh et al. applied this same technique in the cervical spine to prevent non-target embolization via known deep cervical-vertebral-spinal artery connections (31), a principle equally important in the intracranial setting where external-internal carotid communications pose similar risk.

### Calvarial involvement and tumor origin

The precise site of origin of the intracranial lesion in this case warrants consideration. Imaging demonstrated focal osteolytic erosion of the adjacent calvarium with partial extension of the mass through the cranial defect into the overlying subgaleal soft tissues, and intraoperative findings confirmed that the mass was tightly attached to and infiltrating the underlying bone, occupying epidural, dural, and subdural compartments. This pattern raises the possibility of a calvarial bone metastasis with secondary intracranial and extracranial extension, though a primary intracranial lesion with secondary calvarial involvement cannot be excluded. This diagnostic ambiguity is not unique to our case. Chen et al. noted that in their review of intracranial pheochromocytoma metastases, skull infiltration was present in the majority of cases, and that distinguishing between primary skull metastasis with intracranial extension and primary intracranial metastasis with secondary bony involvement is frequently impossible (18). Grabel et al. described a strikingly similar presentation, a painless scalp mass overlying an osteolytic calvarial defect with a large intracranial component supplied exclusively by external carotid artery branches, and found the dura to be completely intact at surgery, which they interpreted as favoring a calvarial origin (28). The precise sequence of spread in our case cannot be determined retrospectively in the absence of earlier interval imaging.

## Limitations

The major limitation of this report is that, as a single case, we cannot definitively isolate the contribution of embolization from excellent anesthetic management alone. A prospective registry comparing embolized versus non-embolized cases would be valuable, though the rarity of this entity makes such a study challenging. Furthermore, as formal germline genetic panel testing was not performed, the precise molecular driver underlying this patient’s multifocal and intracranial metastatic disease cannot be determined.

## Conclusion

This case represents the first documented use of preoperative embolization for intracranial pheochromocytoma metastasis. Complete resection of a 7.8 × 6.0 cm multi-compartmental lesion was achieved without hemodynamic complications through coordinated multidisciplinary planning, alpha-blockade, and targeted embolization. While the specific contribution of embolization cannot be isolated in a single case, our favorable outcome supports its consideration as an adjunct to pharmacologic preparation for functional intracranial pheochromocytoma metastases regardless of tumor size or degree of vascularity.

## Data Availability

The raw data supporting the conclusions of this article will be made available by the authors, without undue reservation.
